# Supplementary material for: Splice-Junction-Based Mapping of Alternative Isoforms in the Human Proteome
Source: Cell Rep. Author manuscript; Available in PMC 2020 Jan 15. (PMC6961840; doi:10.1016/j.celrep.2019.11.026)
Supplement: 2 [file NIHMS1546469-supplement-2.zip › DF1/PXD006675/CardiacFibroblast/CardiacFibroblast_8_SRRM1_KVELSESEDK.pdf]

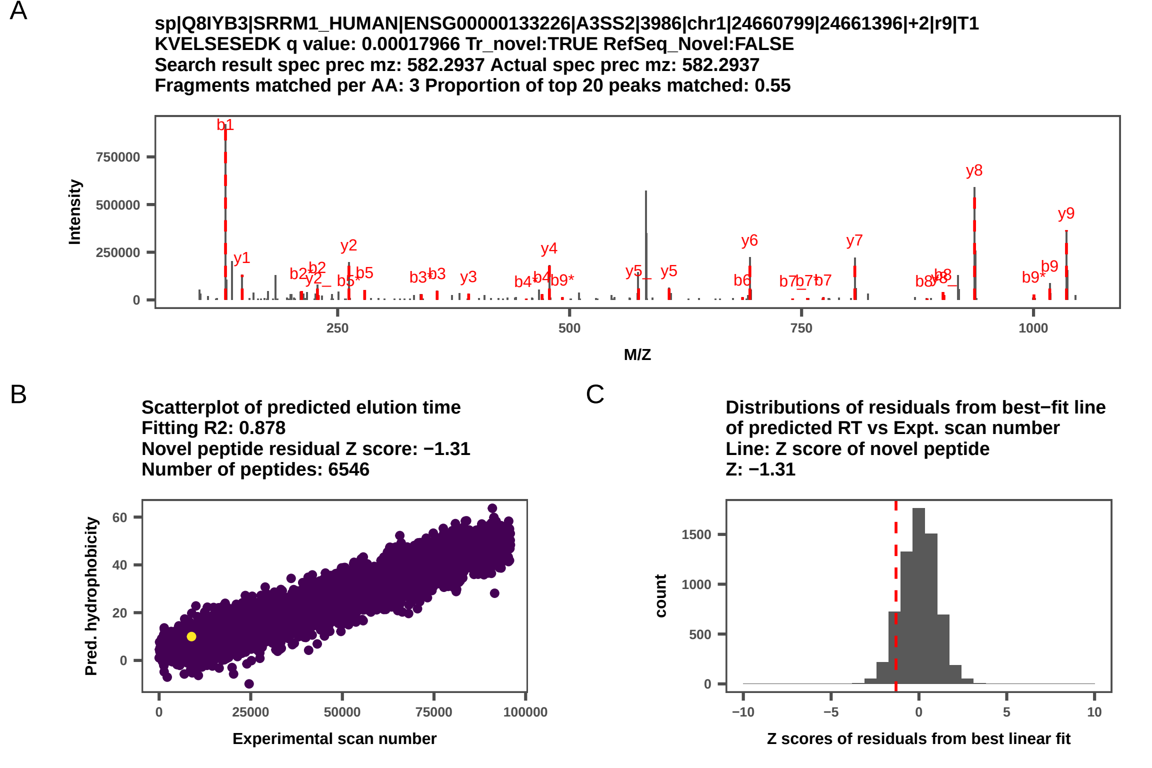
A
sp|Q8IYB3|SRRM1_HUMAN|ENSG00000133226|A3SS2|3986|chr1|24660799|24661396|+2|r9|T1
KVELSESEDK q value: 0.00017966 Tr_novel:TRUE RefSeq_Novel:FALSE
Search result spec prec mz: 582.2937 Actual spec prec mz: 582.2937
Fragments matched per AA: 3 Proportion of top 20 peaks matched: 0.55
Intensity
750000
500000
250000
0
250
500
750
1000
M/Z
b1
y1
b2*
b2
y2_
y2
b5*
b5
b3*
b3
y3
b4*
b4
b9*
y4
y5_
y5
b6
y6
b7_
b7*
b7
y7
b8*
y8_
b8
y8
b9*
b9
y9
B
Scatterplot of predicted elution time
Fitting R2: 0.878
Novel peptide residual Z score: −1.31
Number of peptides: 6546
Pred. hydrophobicity
60
40
20
0
0
25000
50000
75000
100000
Experimental scan number
C
Distributions of residuals from best−fit line
of predicted RT vs Expt. scan number
Line: Z score of novel peptide
Z: −1.31
count
1500
1000
500
0
−10
−5
0
5
10
Z scores of residuals from best linear fit
